# My Way of Treating Nerve Cases

**Published:** 1888-05

**Authors:** Theodore F. Chupein

**Affiliations:** Philadelphia, Pa.


### MY WAY OF TREATING NERVE CASES.

Paper read before the Pennsylvania Association of Dental Surgeons.

BY THEODORE F. CHUPEIN, D. D. S., PHILADELPHIA, PA.

When I speak of "Nerve cases" I include all those cases wherein the nerve or pulp is involved. I do not know, in what I have to say on this subject, whether I have anything new to advance, or that my way of operating has any features of originality, so that those who may be disposed to read this may only be going over the beaten track, yet the learning of some little point of difference in the manner of doing the same thing is a sufficient excuse or incentive for offering an article on a hackneyed subject.

As to the capping of exposed pulps, or pulps nearly exposed, I may say that persistent efforts at this operation for many years past has rather tended to take the starch out of my creed in it. I believe that many cases of what have been regarded as successful operations in this line are successes founded in the imagination or belief of the operator, and not on good grounds; or that they exist in those cases of remarkable constitutions, with a cat-like tenacity to life in the pulp, which we seldom see or have to work for in these days of degenerate health. We have all experienced isolated cases of pulp exposure, when the persistent application of arsenic to the bare tissue has failed to devitalize, and cases are on record in which arsenic has been picked into the pulp, and even this has failed to devitalize. Cases like these might be capped successfully, and life insurance companies could take extraordinary risks, and still make money, on pulps of this kind; but in the very large majority of cases of pulp exposure, pulp capping may be regarded in the same light in which Dr. Hodgkins regards cohesive foil—"a delusion and a snare." I have come to the belief that when a tooth has ached for an hour or more, and that this aching has occurred on two, three or four occasions, that the shortest road to the end is to devitalize and make no attempt to cap, unless, as I said, the patient is one of a robust, vigorous, strong, healthy constitution, which promises the best chances for the success of the operation of pulp capping. If attempted otherwise the operation will surely reap failure, or if successful the success will be short-lived, and he will in all probability have to remove the capping, devitalize and fill roots and crown. I am, therefore, not enthusiastic about capping pulps, and believe that my patients' interest as well as their comfort is better served by root and crown filling.

When the pulp is to be destroyed, if the tooth is giving pain I cleanse out the cavity carefully and apply as much acetate of morphia as will lie on the end of the small blade of a pocket knife, mixed into a paste with oil of cloves, eugenol, carbolic acid or creosote. I apply this in the cavity and seal it with a pellet of cotton. This will ordinarily soothe the pain and keep the tooth comfortable for twenty-four hours or more, and bring it into a better condition for the application of the arsenic. At the next appointment I apply the arsenic. Whenever I can possibly do it, I apply the rubber-dam before applying the arsenic, unless the cavity is easily accessible, or is in the crown surface of a molar or bicuspid, or is in such a position that the sealing plug will not cause the arsenic to ooze out. I always aim at making the application to the actually exposed pulp. In doing this I do not merely apply it to the point that I see is actually exposed, but I probe it in until I see the patient wince or flinch. I feel then that the arsenic will do its work effectually. Sometimes the pain is so severe that I cannot, with the first application, apply the arsenic to the actually exposed pulp. In such cases I rely on the first application for the obtunding effect of the drug. The next application may be made directly to the exposure, as the septum of decayed dentine may be almost painlessly removed to enable me to do this. I leave the arsenic in the tooth from one week to ten days—the text books to the contrary notwithstanding.

The application of arsenic has given me so much trouble from the intolerable pain which it gave my patients, that I have as often shrunk from applying it as they have to have it applied. I have spoken on this subject at different dental societies, only to hear men say "they never have experiences of that kind," but I regard such language in the same light as we regard those operators who never have failures.

Before Dr. Jas. Truman and Dr. E. C. Kirk made their experiments with arsenic, combining it with other drugs for the purpose of making it a painless devitalizing agent, I had sent out to England and procured through Messrs. Ash & Sons, of London, some of "Baldock's improved nerve destroying paste." This preparation guaranteed the painless destruction of the pulp, and I must say that in my hands it has met all requirements, and has been used by me with the most gratifying results. Nearly all the patients for whom I have used it testify in its favor; some to no pain, others to but slight pain, and very few to a pain, or uneasiness extending to more than four hours; in no case was the pain violent. On account of the success I have had with this preparation, I have not tested either Dr. Truman's or Dr. Kirk's formulas.

The pulp being destroyed, my next procedure is to open freely, fairly and directly into the pulp chambers and roots. I do this generally with corundum points on a mandrel secured in the hand-piece of the dental engine. I do not make use of the corundum points which are sold for this and other uses, but make what I want for the case in hand, from small pieces of old broken corundum disks, by heating the mandrel in the blaze of the spirit lamp, coating it slightly, while hot, with gum shellac, and attaching to this small pieces of broken disks. These are in turn kept hot by passing through the blaze, and then rendered cylindrical by manipulating with the fingers, or rolling on the bracket table until they take the form of a fissure bur, or an inverted cone bur. With this, kept well moistened with the drop tube, I cut away all weak points of the crown to secure me a free entrance into the pulp chamber and roots. The borders of the cavity being thus prepared, my next procedure is to apply the rubber-dam. I secure this, not only over the tooth I am to work on, but over two or three others, so that my sight and manipulation may be untrameled. In the use of the rubber-dam I avoid the help of clamps as much as possible. I may use clamps to aid me in applying the dam, but I generally remove the clamps after I have secured the dam with ligatures. Where there is a strain on the dam which might cause it to slip off the tooth, I make a very large knot in the ligature so as to act as a shoulder or stay to prevent the dam from being pulled away from the tooth, and I put smaller knots in each ligature that passes around the teeth. These knots permit the dam to be pressed well up on the necks of the teeth. I place these knots on the lingual or palatal surfaces of the teeth, and tie the ligatures on the labial surfaces. The dam being secured, I first make my entrance into the nerve-chamber with excavators, removing all loosened decayed dentine overlying it. I next clear my way further with a large oval bur in the dental engine, should the case be an upper molar, stopping from time to time to blow out the debris made by the bur with the chip blower, or cleaning out what cannot be blown out with instruments suitable for the purpose. The pulp-chamber being freely entered, I direct my attention next to the roots.

It will often be found that although the pulp may be destroyed there is considerable pain experienced if the effort be made to remove it from the roots. We can account for this in no other way than that the arsenic may have devitalized to a certain point, but beyond this point the pulp is alive, and it is the forcing of the dead part of the organ on to the living, in the effort of removal, or the severing of the living from the dead portion, which causes pain. To keep up this infliction for an indefinite period, giving considerable pain at each thrust of the barbed broach for the removal of the devitalized part, is a procedure that few are disposed to put up with, and I have found that the quickest way for the operator and the most satisfactory one for the patient is the operation known as "knocking out the pulp." This operation consists in sharpening a piece of orange-wood, by whittling it and filing it down to a very fine attenuated point, approximating in length and size to the size of the pulp to be removed. The approach having been made accessible, the point is dipped in carbolic acid. It is then introduced into the root where the pulp is to be removed, and gently insinuated therein until it holds its position. Sometimes it has to be held with the left hand in position, but it is better when it can be introduced sufficiently far to keep its position itself without being held, than to hold it with the left hand. A quick, sharp, decided blow is given to the protruding end of the stick with a small mallet in the right hand, and the operation is completed. Patients report ordinarily little or no pain, others severe, but over so quick that it is done before they have time to complain, and the large majority who admit severe pain prefer this short operation to the tantalizing one of fishing out the pulp with the barbed broach. Generally the pulp, which is completely crushed or mashed by the stick, comes away when the stick is withdrawn, but when it does not, it can be painlessly removed with the pulp canal cleansers used for the purpose. Should the stick break off in the root, it may be easily drilled out, or it may be left in as a root filling, there being sufficient carbolic acid absorbed by the stick to render antiseptic so much of the pulp as may have been pressed between it and the walls of the root canal.

There are many operators who contend that the roots should never be reamed out with pulp canal reamers, and say they should be left as they are made by nature, only cleansing them thoroughly. I cannot, however, conceive how this thorough cleansing can be accomplished unless room is made to do the cleansing, and for this reason I use pulp canal reamers to assist in this part of the work.

If medicines are used for the cure of disease in the roots of teeth, there must be space to make the application of these medicines, and hence the reaming out of the roots is admissible and proper. Of the many instruments put on the market by dental instrument manufacturers, I regard none so highly as the Morey nerve canal reamers. These were once manufactured by the S. S. White Dental Manufacturing Co., and the instruments made by them were a pleasure to work with. They were keen, well tempered, each leaf of the cutting head the same size and on a level with its fellow, and the shaft just flexible enough in proportion to the size of the working points. But since they have discontinued making them, I have never been able to procure from any instrument maker any of these reamers so admirably adapted for the work as those I first obtained from the White Company. Some operators have told me that they break many of these drills in the roots, but I can assure my listeners that I have only had one accident of this kind happen to me after perhaps two or three years' use of these instruments. The accident, I think, is likely to occur from the directions that are given for their use. We are told that the smallest size should be used first, gradually increasing as progress is made. Now my way of procedure is just the reverse. I use the largest size as I enter the root, and ream out with this to a certain depth, cleansing the debris constantly as I proceed; then I use a size smaller and proceed in the same way with this, and finally with the smallest size. I use only three sizes. If there is any choking of the canal it is needless to expect these instruments to make way through the obstruction; they will not do it, for they are reamers, not drills. I free the obstruction with the smallest probes, and when the probe enters the canal the reamers will follow and make clean work. The probes I make myself, out of piano wire, by securing a piece in a suitable handle and cutting it down gradually on a corundum stone in the polishing lathe, and afterwards bringing it down to the finest point with emery paper. A probe of this kind possesses all the flexibility necessary for the work expected of it, and is sufficiently tough never to break off in the canal, except by very rough or careless usage.

It will be found that in many roots the entrance or opening is not sufficiently cylindrical to permit the use of the reamers at the offset. They are uneven and ragged, and if force is used to make the reamers cut the chances are that they may be broken by the force used. I overcome this difficulty by first preparing the way by the use of a flexible bur, such as is known as 160 of the S. S. White Dental Catalogue for 1876, in the list of instruments for the dental engine. This instrument smoothes down the angles at the opening and prepares the way for the reamers to follow without liability of breaking. The roots being all cleaned out by the use of the reamers and other instruments used for the purpose, are then filled. If the case is one of pulp devitalization, the roots and crown may be filled at once, but if it be one of putrescent pulp or alveolar abscess, I treat the roots by medication and disinfection before final filling. I fill the roots with cotton. I have been using cotton as a root-filling for twenty-five years, and though I have tried the other materials suggested, I have not been able to see that any of them possess advantages over a root-filling of cotton. With the roots prepared for filling as I have described, it is quite as easy to fill them with wood, chloride or phosphate of zinc, gold, tin, lead or gutta-percha, yet, as I have said, I cannot see, nor has experience taught me, that any of these materials are any better than cotton. I introduce the cotton in very small pieces, about the size of a mustard seed bird-shot, saturated with creosote or carbolic acid, or with an ethereal solution of iodol, and not in a long string, as is generally done. Introduced in this way the cotton can be carried up as far as the reamers have prepared the root, without choking, which is not always possible with cotton made into a long thread or string. The roots filled, I fill the crown with gold or amalgam, as seems best for the case.

If I have a pulp case with an external fistula to deal with, I do not put on the rubber dam until a cure is effected. I pursue the same treatment in these cases, in the matter of reaming out the roots, removing all debris as I proceed. Before medicating the roots I make sure that the canal is open at the apex, by using the probe freely and judging the length of the root by the depth that the probe passes into the root, as also by the flinching of the patient when the probe passes through the foramen. I use as medicines, 1st, the bichloride mercury (1 to 1000) freely on a probe to which a few shreds of cotton are attached, pumping this in. If there is much pus I use after this the peroxide of hydrogen in the same way. After this I pump creosote or carbolic acid through the root along the track of the abscess, until it bubbles or froths at the fistulous opening*. I have had such good success with either of these two old remedies for the treatment of abscess, that I have great reliance on them. If these medicines refuse to pass through the root and appear at the fistulous opening, it is a sign that the apex of the root is not freely opened, or it may be that the end of the root is crooked, as is frequently the case with the ends of the roots of the lateral incisors and the roots of the first bicuspids. If the root is crooked nothing can be done to free it, and all that may be done is to treat the abscess from the outside. I never feel as confident of a cure when the abscess is treated through the external fistula as when medicines are carried through the root and along the track of the abscess. If the tooth be a central incisor, a cuspid, a second bicuspid or the palatine root of an upper molar, the failure of medicines to pass through will doubtless be due to debris choking the foramina of these roots. This must be cleared away with probes or broaches, otherwise a cure cannot be looked for. It is the action of these medicines on these diseased surfaces which effect the cure, and if the medicines do not come in contact with them a cure cannot be expected.

It is well to examine your probes and broaches from time to time with a strong magnifying glass, as there may be flaws in them which may not affect them at first, but which may cause them to break after constant use, and as will be most likely, to break in the root, causing the operator a world of trouble to remove. When such flaws are discovered it is best to discard the instrument rather than run the risk of a fracture in the root.

The abscess being treated as described, the root is filled lightly with cotton; the orifice of the cavity is also lightly sealed against the ingress of small particles of food. If on the return of the patient things look favorable, the root may be filled tightly with cotton and the orifice also closely sealed. If unfavorable a renewal of the medication is indicated. On the third presentation, if on the removal of the cotton it is found clean, free from stain or odor, the rubber dam may be applied and the tooth filled. The roots

---

* Would it not be better to use the detergent (peroxide of hydrogen) first, and follow this with the germicide or antiseptic? We cannot comprehend the reason for the employment of the mercuric chloride and carbolic acid both. Creosote, not being of the same nature, is never indicated where carbolic acid is.—EDITOR.

should be made quite dry before filling. If the case is one of a central or lateral incisor or a cuspid, where the entrance to the root is made through the palatal aspect of the teeth, I prefer to fill the cavity (after filling the root) with gutta-percha, and let the case go thus for a year or more. There is little or no wear on the gutta-percha on these surfaces, and should there be any after trouble (which might occur when all things looked favorable), it is easy to remove the gutta-percha and renew the treatment. All good soldiers look to an easy retreat. In bicuspids and molars, where the approach is made from their disto-masticating or mesio-masticating aspects, I fill the roots and cover the floor of the cavity with gutta-percha, letting this extend by gradual manipulation to a feather edge at the cervical margin of the cavity, and over this I fill with phosphate cement. Should the case give trouble after a year's probation, it is easy to remove the filling and renew the treatment to give relief.

In cases of putrescent pulp, I get rid of the odor by first washing out with Eau de Cologne.* I then apply the dam and use bichloride freely, and next the peroxide of hydrogen. I disinfect with a saturated solution of iodol in ether. I have been using this in preference to iodoform because of the disgusting and persistent odor of the latter. In conversation with a physician who has quite a large practice, for whom I was treating a tooth, he informed me that iodol was much more extensively used as a disinfectant among physicians than iodoform. My success with it, as far as I have been able to observe, has been very marked. If there be any truth in the quotation "*Similia similibus curantur,*" then iodol ought to cure putrescent pulp, for if a probe be dipped in the solution and then put into the blaze of a spirit lamp it will give off an odor almost identical with the odor we are all familiar with as coming from putrescent pulps. Try it.

In immediate root-filling I have not taken much stock, yet I think when a dry, powdery condition of the pulp is observed, or where the pulp has been devitalized and the pulp all removed and the roots properly treated and disinfected, the case may be permanently filled, but I do not think this should ever be done in cases of alveolar abscess or putrescent pulps.

---

* Not a disinfectant.

